# Brazilian Dialysis Survey 2021

**DOI:** 10.1590/2175-8239-JBN-2022-0083en

**Published:** 2022-11-04

**Authors:** Fabiana B Nerbass, Helbert do Nascimento Lima, Fernando Saldanha Thomé, Osvaldo Merege Vieira, Ricardo Sesso, Jocemir Ronaldo Lugon

**Affiliations:** 1 Fundação Pró-Rim, Joinville, SC, Brazil.; 2 Universidade da Região de Joinville, Joinville, SC, Brazil.; 3 Universidade Federal do Rio Grande do Sul, Porto Alegre, RS, Brazil.; 4 Universidade de São Paulo, Ribeirão Preto, Brazil.; 5 Universidade Federal de São Paulo, São Paulo, SP, Brazil.; 6 Universidade Federal Fluminense, Niterói, RJ, Brazil.

**Keywords:** Renal Dialysis, Peritoneal Dialysis, Epidemiology, COVID-19, Diálise Renal, Diálise Peritoneal, Epidemiologia, COVID-19

## Abstract

**Introduction:**

The Brazilian Dialysis Survey (BDS) is an important source of national data about people on chronic dialysis that contributes to the formulation of health policies regarding kidney failure.

**Objective:**

To report the 2021 epidemiological data from the BDS of the Brazilian Society of Nephrology (BSN).

**Methods:**

A survey was carried out in Brazilian chronic dialysis centers using an online questionnaire covering clinical and epidemiological aspects of patients in chronic dialysis, data on dialysis therapy, characteristics of dialysis centers, and the impact of the COVID-19 pandemic of 2021.

**Results:**

Thirty percent (n = 252) of the centers answered the questionnaire. In July 2021, the estimated total number of patients on dialysis was 148,363. The estimated prevalence and incidence rates of patients per million population (pmp) were 696 and 224, respectively. Of the prevalent patients, 94.2% were on hemodialysis (HD) (1.8% of these on hemodiafiltration), and 5.8% on peritoneal dialysis (PD); 21% were on the transplant waiting list. The incidence rate of confirmed COVID-19 between January and July 2021 was 1,236/10,000 dialysis patients, and the case-fatality rate reached 25.5%. Up to July 2021, 88.6% of patients had received at least one dose of the anti-SARS-CoV-2 vaccine. The estimated overall and COVID-19 crude annual mortality rates were 22.3% and 5.3%, respectively.

**Conclusion:**

The absolute number and the prevalence rate of patients on chronic dialysis continue to increase. Most dialysis patients were vaccinated against COVID-19 during the year. The COVID-19 pandemic was associated to the overall mortality rate.

## RESUMO

**Introdução:**

O Censo Brasileiro de Diálise (CBD) constitui importante fonte de dados nacionais sobre pessoas em diálise crônica que contribui para a formulação de políticas de saúde.

**Objetivo:**

Relatar dados epidemiológicos de 2021 do CBD da Sociedade Brasileira de Nefrologia (SBN).

**Métodos:**

Realizou-se pesquisa em centros brasileiros de diálise crônica utilizando questionário online abrangendo aspectos clínicos e epidemiológicos de pacientes em diálise crônica, dados sobre terapia dialítica, características dos centros de diálise e o impacto da pandemia COVID-19 em 2021.

**Resultados:**

Trinta por cento (n = 252) dos centros responderam ao questionário. Em Julho de 2021, o número total estimado de pacientes em diálise foi 148.363. As taxas estimadas de prevalência e incidência de pacientes por milhão da população (pmp) foram 696 e 224, respectivamente. Dos pacientes prevalentes, 94,2% estavam em hemodiálise (HD) (1,8% destes em hemodiafiltração), e 5,8% em diálise peritoneal (DP); 21% estavam na lista de espera para transplante. A taxa de incidência de COVID-19 confirmada entre Janeiro e Julho de 2021 foi 1.236/10.000 pacientes em diálise, e a taxa de letalidade atingiu 25,5%. Até Julho de 2021, 88,6% dos pacientes haviam recebido pelo menos uma dose da vacina anti-SARS-CoV-2. As taxas estimadas de mortalidade bruta anual geral e por COVID-19 foram de 22,3% e 5,3%, respectivamente.

**Conclusão:**

O número absoluto e a taxa de prevalência de pacientes em diálise crônica continuam aumentando. A maioria dos pacientes em diálise foram vacinados contra COVID-19 durante o ano. A pandemia COVID-19 contribuiu com a taxa de mortalidade geral.

## Introduction

The Brazilian Society of Nephrology (BSN) promotes an annual survey to collect and analyze trends in epidemiological and clinical aspects of patients undergoing chronic dialysis in our country. Since 1999, the Brazilian Dialysis Survey has provided important information for the development of health policies and strategies aimed at improving the care of thousands of individuals undergoing chronic dialysis treatment in our country.

In this manuscript, we report the main results of the 2021 Brazilian Dialysis Survey, which included information about the impact of the COVID-19 pandemic on patients and staff of dialysis clinics.

## Methods

### Data Collection

Dialysis centers filled out an online questionnaire available on the BSN website. It contained questions about sociodemographic, clinical, and therapeutic parameters of patients on chronic dialysis and was available from August 2021 to January 2022. Participation in the survey was voluntary, and all dialysis centers registered at BSN were invited by email and BSN media to participate. After the initial invitation, new reminders were sent monthly to centers that had not informed their data. During the survey period, BSN regional presidents were asked to contact the dialysis centers in their states, to reinforce the importance of participation.

### Data Analysis

The data for each center were provided grouped rather than individually. For the 2021 survey, 252 out of 849 active centers answered the questionnaire, corresponding to a response rate of 30%.

The sample was expanded for national estimates of the total number of patients and prevalence rate. We considered that the units that did not answer the questionnaire had the same number of patients as the participants (mean of 175 patients per unit). As this extrapolation can be imprecise, we used a variation of ±5% in the obtained mean (166 to 183 patients per unit) for prevalence calculations. Likewise, the mean number of new patients per center was applied to centers that did not inform incidence rates. All other sociodemographic data and patient characteristics pertain to the studied sample. The annual mortality and annual incidence of patients on dialysis were estimated from the occurrences of July 2021. For calculating the prevalence and incidence rates, national and regional population data were obtained from the Brazilian Institute of Geography and Statistics (IBGE) estimates for July 2021. According to IBGE, the Brazilian population was 213.32 million inhabitants^1^. Most data were descriptive, and the results were compared with data from previous years. Regarding information on COVID-19, such as incidence, hospitalization, and lethality, the period considered was from January 1^st^ to July 31^st^, 2021. Diagnosis of COVID-19 required confirmation by real-time polymerase chain reaction (RT-PCR) of nasal/oropharynx specimens or serology.

### Calculations Performed for Estimates

The main calculations and estimates are shown in Table 1.

**Table 1.** Calculations of estimates of incidence, prevalence, and mortality

Estimates	Formula
Estimated total number (N) of patients on 1^st^ of July	N of patients in the sample / proportion of participating centers
Estimated annual prevalence rate of dialysis patients (pmp)	Estimated total N of patients on 1^st^ of July / Brazilian population on 1^st^ of July^1^
Estimated total N of patients starting treatment	N of individuals starting treatment in July × 12 / proportion of active participating centers
Estimated annual incidence rate of dialysis patients (pmp)	Estimated total N of patients starting treatment / Brazilian population on 1^st^ of July^1^
Estimated total annual N of deaths	N of deaths reported in July × 12 / proportion of active participating centers
Estimated crude annual mortality rate (%)	Estimated total N of deaths in 2021 × 100 / estimated N of dialysis patients on 1^st^ of July
Estimated COVID-19 crude annual mortality rate (%)	N deaths due to COVID-19 from January to July 2021 × (12/7) × 100 / estimated N of dialysis patients on 1^st^ of July

pmp: per million population.

## Results

### Estimated Incidence, Prevalence, and Mortality Rates

In July 2021, there were 849 active chronic dialysis centers registered at BSN, 1.8% higher than in 2020. In the whole country, there were four dialysis centers per million population (pmp), with lower rates in the North (2.7 pmp) and Northeast (2.8 pmp) regions compared with the Southeast (4.5 pmp), Mid-West (4.7 pmp), and South (5.0 pmp) regions.

The percentage of participating centers was slightly higher compared to the previous year (from 28% to 30%). The region with the highest participation was the South (39%), followed by Middle-West (31%), Southeast (28%), North (27%), and Northeast (26%). The number of patients in the current BDS was 8% higher than in 2020 (from 40,795 to 44,037).

**Figure 1.**
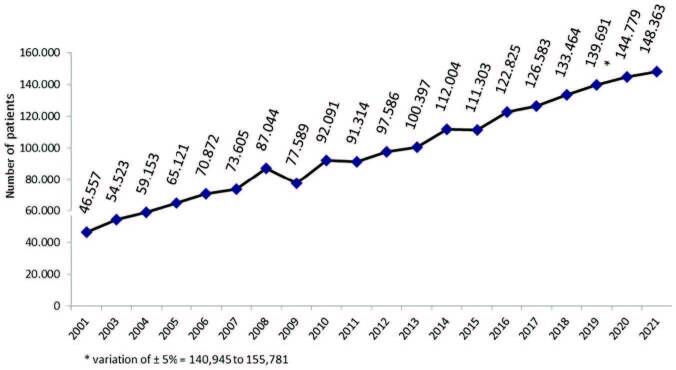
Estimated number of patients on chronic dialysis per year.

**Figure 2.**
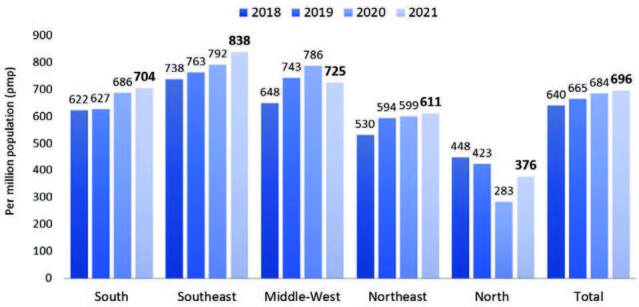
Estimated prevalence rate of patients on dialysis by geographic region in Brazil, per million population.

**Figure 3.**
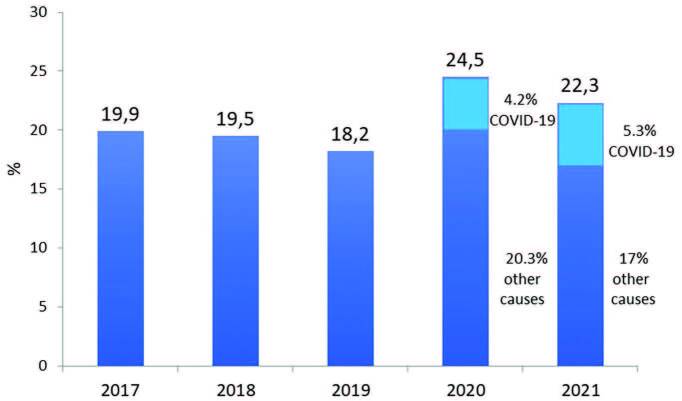
Estimated annual crude mortality rate of dialysis patients.

The estimated total number of patients in July 2021 was 148,363 (variation of ±5% = 140,945 to 155,781), 2.5% higher than in July 2020. The trend toward an increment in number of patients on dialysis observed in recent years persisted in 2021 (Figure 1). The prevalence rate of dialysis patients also continued to rise, from 684 pmp in 2020 to 696 pmp in 2021. When stratified by region, a significant decrease in prevalence rate was only observed only in the Middle-West (Figure 2). The estimated number of new dialysis patients in 2021 was 47,886. The overall incidence rate was 224 pmp, higher than in 2020 when it reached 209 pmp, ranging from 117 pmp in the North to 272 pmp in the Southeast. The estimated number of deaths in the whole year was 33,101. The annual crude mortality rate decreased from 24.5% in 2020 to 22.3% in 2021 (Figure 3).

#### Demographic and clinical characteristics

**Figure 4.**
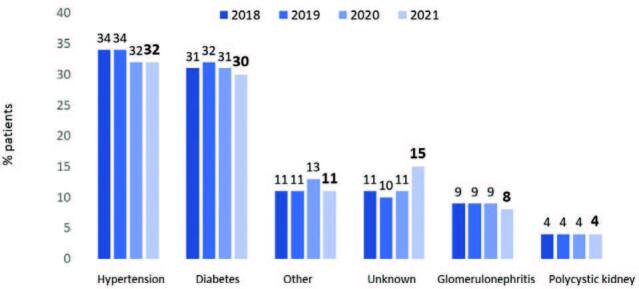
Distribution of dialysis patients according to chronic kidney disease etiology.

**Figure 5.**
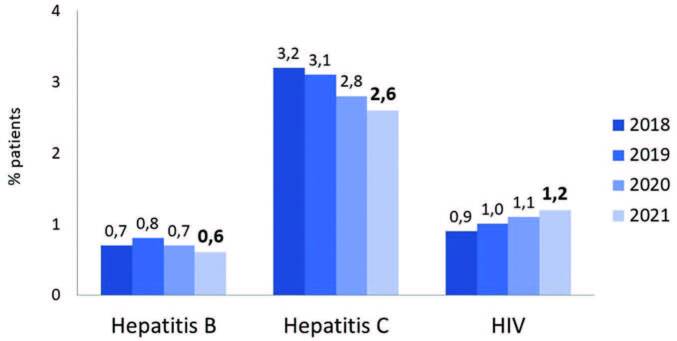
Prevalence of patients with positive serology for hepatitis B and C and HIV.

**Figure 6.**
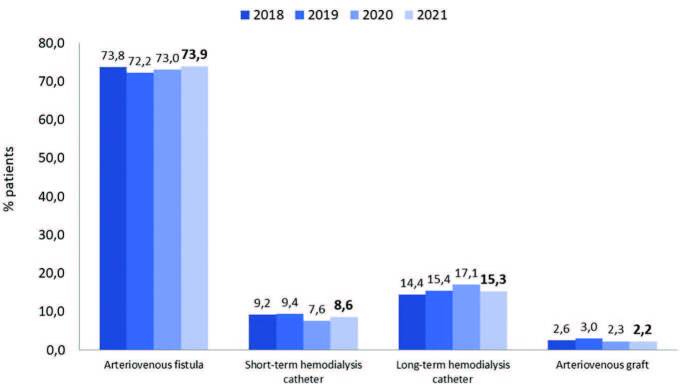
Type of vascular accesses used for hemodialysis.

Sex distribution of 59% (n = 25,352/43,176) male and 41% (n = 17,824/43,176) female remained stable, and so did the percentage of the main underlying diseases. Systemic arterial hypertension and diabetes mellitus represented almost a third of all cases each (Figure 4). The percentage of patients with hepatitis B (0.6%; n = 280/44,037) and C (2.6%; n = 1,142/44,037) continued to decline, while that of patients with HIV increased slightly (1.2%; n = 511/44,037) (Figure 5). Regarding vascular access for hemodialysis (HD) patients, 23.9% (n = 10,533/41,457) used a central venous catheter. The use of long-term catheters decreased, accompanied by an increase in short-term catheters and arteriovenous grafts (Figure 6). The estimated number of dialysis patients on the kidney transplant waiting list in 2021 was 30,439 (21%), lower than in the previous year (23%).

#### Characteristics of dialysis treatment

**Figure 7.**
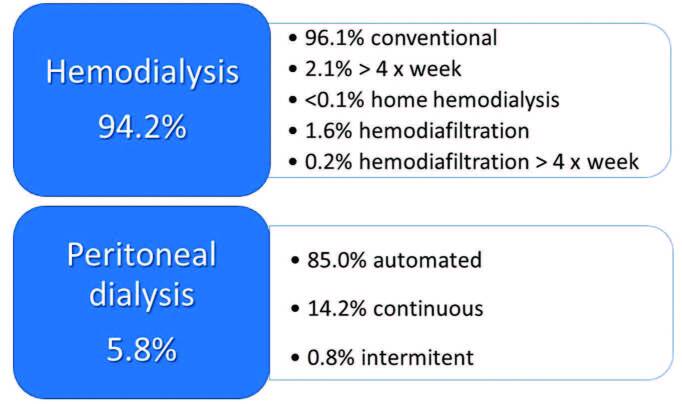
Distribution of patients according to dialysis modality.

The distribution of patients according to dialysis modality and funding source are shown in Table 2 and Figure 7. The public health system was the funding source for 81.8% (n = 36,040/44,037) and private health insurance funded 18.2% (n = 7,997/44,037) of patients. Regarding dialysis modality, hemodialysis (HD) was the most common treatment and increased from 92.6% to 94.2% in 2021. Among the HD patients, the vast majority were on conventional HD (96.1%; n = 39,856/41,457), 2.1% (n = 873/41,457) were on HD >4x/week, 1.8% (n = 712/41,457) were on hemodiafiltration, and less than 0.1% (n = 16/41,457) were on home hemodialysis. Of the 5.8% (n = 2,580/44,037) patients on peritoneal dialysis (PD), 85% (n = 2,194/2,580) were on automated peritoneal dialysis (APD).

**Table 2.** Distribution of patients by modality of dialysis and paying source

Modality	Public health			Private health			Total	
	N	%	N		%	N		%
HD ≤ 4 sessions/week	33,880	94.0	5,976		74.7	39,856		90.5
HD > 4 sessions/week	136	0.4	737		9.2	873		2.0
Home HD	0	0.0	16		0.2	16		0.0
HDF ≤ 4 sessions/week	47	0.1	592		7.4	639		1.5
HDF > 4 sessions/week	5	0	68		0.9	73		0.2
CAPD	315	0.9	50		0.6	365		0.8
APD	1,636	4.5	558		7.0	2,194		5.0
IPD	21	0.1	0		0	21		0.0
**Total**	36,040	100	7,997		100	44,037		100

HD: hemodialysis; HDF: hemodiafiltration; CAPD: continuous ambulatory peritoneal dialysis; APD: automated peritoneal dialysis; IPD: intermittent peritoneal dialysis.

#### Characteristics of participating centers

Of the 252 participating dialysis centers, 73.8% were privately owned, 18.3% were philanthropic, and 7.9% were public. International corporations managed 17.9% of participating units among the privately owned ones. Most centers identified themselves as satellite centers (57%), and in-hospital centers comprised 43%. The national number of patients per nephrologist was 30, ranging from 26 in the North to 37 in the South.

#### Covid-19

**Figure 8.**
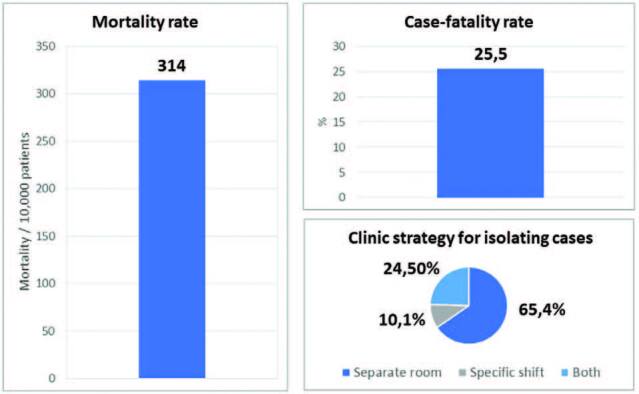
COVID-19 mortality rate, case-fatality rate, and strategy adopted to isolate cases.

Between January and July 2021, there were 5,344 COVID-19 reported cases and 1,362 deaths. The incidence rate of confirmed COVID-19 was 1,236/10,000 dialysis patients; the case-fatality rate was 25.5%; the mortality rate reached 314/10,000 patients. The estimated crude annual mortality rate attributed to COVID-19 was 5.3%. Up to July 2021, 88.6% of dialysis patients had received at least one vaccine dose. As a strategy for isolating suspected or confirmed cases of COVID-19, 65.4% of the centers reported providing treatment in a separate room or space, 10.1% adopted transfer to a specific ward, and 24.5% used both strategies (Figure 8). The confirmed infection percentage of health professionals working in clinics was 26.9%. Infected physicians, nurses, and nursing technicians were 24.9%, 26.8%, and 27.4%, respectively. Three deaths by COVID-19 were reported, one in each occupation.

## Discussion

For the last two decades, the BDS has been offering a panorama of dialysis treatment, providing data and analyses that contribute to the development of public policies and strategies to improve this therapy in our country. In general, the trends observed in recent years were maintained in 2021. In addition, as a novelty, we reported the prevalence of patients on hemodiafiltration and the COVID-19 vaccine coverage.

The percentage of dialysis centers participating in 2021 increased slightly compared to the previous year, from 28% to 30%^2^. The upward trend in the total number of patients by 2.5% and the prevalence by 1.7% followed the pattern observed across time. However, it was less pronounced than in the past five years, when the mean increase in the number of patients and prevalence was 5.4% and 4.7%, respectively. A possible reason for this finding was the high mortality rate observed during the COVID-19 pandemic^2,3^.

Our prevalence of people on dialysis (696 pmp) was slightly higher than the average of the 2019 Latin American Society of Nephrology (SLAHN) registry (650 pmp)^4^ and the 2019 European registry (562 pmp)^5^. On the other hand, our numbers were substantially lower than those from the United States in 2019 (1628 pmp)^6^. The 2021 incidence rate (224 pmp) was higher than the 2020 national estimate (209 pmp), higher than the Latin America (168 pmp)^4^ and Europe (125 pmp) rates in 2019^5^, and lower than the 2019 United States (392 pmp) rate^6^.

The 22.3% crude mortality rate was lower than last year’s when it reached 24.5%. As in 2020, the high mortality associated with COVID-19 in dialysis^3^ may have contributed to the finding. According to our estimate, the annual crude mortality rate attributed to COVID-19 was 5.3%, corresponding to 23.8% of the overall annual crude mortality rate.

The rate of primary disease diagnosis was stable, with hypertension accounting for 32% and diabetes 30%. Although the precision of these estimates may be questioned due to the difficulty of establishing the primary diagnosis of CKD, we have consistently found that diabetes has not outpaced hypertension as the leading primary disease in our country in prior surveys. In contrast, diabetes has been the main primary diagnosis in the US dialysis population in 2017 (45%), while hypertension represented 30%^7^.

There was a downward trend in the percentage of patients with hepatitis C, reaching 2.6% for the first time. As in previous years, almost a quarter of HD patients (23.9%) used a central venous catheter as the vascular access (8.6% short-term and 15.3% long-term). Almost three-quarters (73.9%) of patients used an arteriovenous fistula, and the remaining 2.2% had an arteriovenous graft. The United States Renal Data System (USRDS) reported that in 2019 18.8%, 64.5%, and 16.8% of prevalent HD patients used central venous catheter, arteriovenous fistula, and arteriovenous graft, respectively. According to the 2020 KDOQI clinical practice guideline for vascular access, there is inadequate evidence for making a recommendation on the preferred type of vascular access in prevalent HD patients based on vascular access outcomes, patient hospitalizations, or mortality. However, the KDOQI considered it is preferable to use arteriovenous access (fistula or graft) to a central venous catheter in prevalent HD patients. Furthermore, if clinical circumstances are favorable, a mature and usable arteriovenous fistula is preferred over an arteriovenous graft in prevalent HD patients^8^.

For the first time, the survey included the number of patients on hemodiafiltration (HDF). The prevalence was 1.8% of all patients on hemodialysis. This rate is far below the global prevalence estimated at 10% in 2018 but close to Latin American numbers (1.5%)^9^.

Although almost half of the participating centers offer PD as a treatment option (48%), only 5.8% of dialysis patients were treated by this modality. The current prevalence in Latin American countries is 14%^4^. There was a significant increase in the predominance of automated peritoneal dialysis (APD) compared to the previous two years (85% versus ∼60%), following a trend observed in developed countries^10^.

Regarding COVID-19, between January 1^st^ and July 31^st^, 2021, we found an incidence rate of infection of 1,236/10,000 patients. Between February 26^th^ to July 31^st^, 2020, the rate was 684/10,000 patients. The time frame for data collection in 2021 was two months longer, but the higher incidence observed in that year can also reflect higher test availability and a real increase in new cases accompanying the second peak of the pandemic in the country. The case-fatality rate of 25.5% was very close to the one observed in a Brazilian dialysis cohort in 2020 (27.7%)^3^ and consistent with global results reported for the dialysis population^11,12,13,14^. The high vaccination coverage rate of patients in July (88.6%) can be considered the result of the high availability of the vaccine and population awareness of the importance of immunization. The introduction of vaccination for dialysis patients in the country was in April 2021.

As limitations, we highlight the electronic data collection through voluntary completion, the aggregation of patient data by dialysis center, and the lack of response validation. Furthermore, due to the participation of approximately 30% of active dialysis centers, the methodology used in national prevalence estimates and incidence rates had limited accuracy, and caution is advisable regarding data interpretation.

In conclusion, the 2021 survey confirmed a continuous increase in the prevalence of dialysis patients over the years. On the other hand, the low prevalence of PD as dialysis therapy remains, although this dialysis modality is offered in most centers. Also, the high case-fatality rate of COVID-19 on dialysis once more played a role in this population’s overall crude mortality rate.
